# The impact of effort–reward imbalance on academic burnout: the chain mediating effect of frustration and perceived stress

**DOI:** 10.3389/fpsyg.2026.1726989

**Published:** 2026-01-21

**Authors:** Hehe Ma, Qiangqiang Wang, Jianglong Shen

**Affiliations:** School of Teacher and Education, Huzhou University, Huzhou, China

**Keywords:** academic burnout, effort–reward imbalance, frustration, mediating effect, perceived stress

## Abstract

**Objective:**

Although effort–reward imbalance and academic burnout were usually experienced by adolescents in their lives, whether and how effort–reward imbalance influences adolescents’ academic burnout remains unclear. Therefore, the present study aimed to investigate the relationship between effort–reward imbalance and academic burnout, as well as the chain mediating effect of frustration and perceived stress between effort–reward imbalance and academic burnout.

**Methods:**

A sample survey was conducted with 1,402 middle school students using the Effort–reward imbalance Scale, the Defeat Scale, the Perceived Stress Scale, and the Academic Burnout Scale. Descriptive statistical analysis and correlation analysis were used with SPSS 26.0 software, and the chained mediation model was tested with the PROCESS 4.1 program.

**Results:**

(1) There is a significant correlation between effort–reward imbalance, frustration, perceived stress, and academic burnout; (2) the direct predictive effect of effort–reward imbalance on academic burnout is significant; and (3) the chain mediating effect of frustration and perceived stress between effort–reward imbalance and academic burnout is significant.

**Conclusion:**

Effort–reward imbalance affects academic burnout through the mediating effects of frustration and perceived stress as well as through the chain mediating effect of frustration and perceived stress. These findings suggest that in school education, we should prevent effort–reward imbalance and its impact on teenagers’ frustration and perceived stress, which is conducive to reducing academic burnout among teenagers.

## 1 Introduction

Academic burnout is a negative psychological state that students develop when they face academic demands and pressure for a long period of time and includes three dimensions: emotional depletion, a low sense of accomplishment, and cynical attitudes (Schaufeli et al., 2002; Wang et al., 2023). Previous research has shown that students’ academic burnout has a significant predictive effect on academic accomplishment. The higher the level of academic burnout is, the lower the degree of academic accomplishment they achieve (Madigan and Curran, 2021; Schaufeli et al., 2002; Wang et al., 2015). Therefore, exploring the factors and underlying mechanisms of student academic burnout has become a hot topic of great concern among educational researchers (Jin et al., 2025; Wang et al., 2023; Wang et al., 2015; Yin et al., 2022). Existing research has explored the mechanism of action of student academic burnout from dimensions such as individual characteristics (Yu et al., 2023), family background factors (Yin et al., 2022), and the school educational environment (Luo et al., 2014). However, few studies have investigated the relationship and mechanism of action between effort–reward imbalance and academic burnout among teenagers. Therefore, this study aims to investigate the predictive effect and mechanism of effort–reward imbalance on academic burnout among teenagers from the perspectives of frustration and perceived stress. Academic burnout is a common phenomenon faced by teenagers in many countries and regions during their studies (Wang et al., 2023; Xu et al., 2022). Investigating how effort–reward imbalance influences academic burnout can clarify psychological mechanisms underlying academic burnout and offer important indications for the design of educational interventions oriented toward the management of effort, the perception of fairness in school rewards, and emotional regulation in contexts of high academic demands.

### 1.1 Effort–reward imbalance and academic burnout

The effort–reward imbalance (ERI) model holds that after individuals invest a certain amount of time, energy, and effort into their work, on the basis of the social exchange contract, they all expect to receive corresponding rewards, such as money, respect, and status, from the organization or environment. When an individual does not receive the expected return after paying a certain cost, a stress response occurs, leading to emotional distress and stress response (Siegrist, 1996). Subsequent research has shown that the effort–reward imbalance model can also be applied to the student group (Jin et al., 2025; Li et al., 2010; Vilser et al., 2024; Wang et al., 2023). For students, effort is reflected mainly in their investment in terms of study time, money, and hard work. The corresponding rewards refer mainly to their eagerness to achieve corresponding improvements in academic accomplishment, esteem from others, and their status (Jin et al., 2025). According to the effort–reward imbalance model, when students experience an effort–reward imbalance during the learning process, they will have a sense of unfairness and continuous pressure, which in turn leads to emotional depletion and powerlessness, as well as indifference and estrangement from the learning content, along with a decline in the sense of accomplishment (Gao et al., 2025; Siegrist, 1996). Emotional depletion, cynical attitudes, and a low sense of accomplishment are precisely the three core dimensions of academic burnout (Schaufeli et al., 2002; Wang et al., 2023). From these findings, it can be inferred that the effort–reward imbalance significantly predicts teenagers’ academic burnout. Several recent empirical studies have also reported that the greater the sense of effort–reward imbalance experienced by students during the learning process, the greater the level of academic burnout (Gao et al., 2025; Jin et al., 2025; Wang et al., 2023). This research further supports the significant predictive effect of effort–reward imbalance on academic burnout among teenagers. On the basis of the above analysis, this study proposes.

*Hypothesis H*1: Effort–reward imbalance positively predicts academic burnout.

### 1.2 Mediating effect of frustration

Frustration is a negative emotional state that occurs when an individual encounters obstacles, disruptions, or disturbances in the pursuit of their goals, resulting in unfulfilled needs and difficulty in achieving goals (Dollard et al., 1939). Frustration theory holds that “goal obstruction” caused by unmet needs is the fundamental mechanism that triggers negative emotions. Frustration arises when external or internal factors prevent an individual from achieving their expectations (Maier, 1956; Miller, 1941). According to the frustration theory, when students invest a certain amount of time, money, effort, and energy in their research but do not receive the returns they expect, such unmet expectations or needs can lead to their “goal being blocked,” which is very likely to trigger their frustration.

Furthermore, self-determination theory (SDT) holds that individuals are innately endowed with three fundamental psychological needs: autonomy, competence, and relatedness. When these needs are met, intrinsic motivation is activated, and individuals experience a sense of self-worth, efficacy, and emotional belonging, thereby maintaining a good state of mental health (Deci and Ryan, 2000; Ryan and Deci, 2000). Conversely, when there is a need thwarting in the learning context, such as when classroom teaching restricts students’ choices, when learning tasks exceed students’ capabilities, or when teacher–student relationships lack support, it triggers feelings of frustration (Vansteenkiste and Ryan, 2013). This sense of frustration is essentially the perception that basic needs have not been met. It consumes an individual’s emotional resources, making them feel powerless, frustrated, and passive. Vansteenkiste and Ryan (2013) reported that demand frustration is the key pathway leading to a series of ill-being states, which can trigger negative experiences, including emotional depletion, disengagement, and reduced efficacy. These experiences precisely correspond to the three core dimensions of academic burnout: emotional depletion, cynical attitudes, and a low sense of accomplishment. Therefore, on the basis of self-determination theory, it can be reasonably inferred that frustration can significantly predict academic burnout among teenagers. Related empirical research has also confirmed that academic frustration has a significant positive effect on academic burnout (Fariborz et al., 2019; Zhang and Jiang, 2023). For instance, Zhang and Jiang (2023) conducted a potential profile analysis of 1,521 Chinese high school students on the basis of self-determination theory and reported that students in the high frustration group (such as the “low-satisfaction - moderate frustration” and “moderate satisfaction - high frustration” profiles) exhibited higher levels in all dimensions of academic burnout, particularly in terms of emotional depletion and learning alienation. Therefore, on the basis of the above analysis, Hypothesis H2 can be inferred:

*Hypothesis H*2: Frustration plays a mediating role between effort–reward imbalance and academic burnout.

### 1.3 Mediating effect of perceived stress

Perceived stress refers to an individual’s subjective assessment of the uncontrollability or difficulty in resolving life events, reflecting the degree of psychological stress experienced by the individual (Cohen et al., 1983). The new ternary effort–reward imbalance model indicates that the effort–reward imbalance experienced by individuals can significantly predict their work stress. When an individual’s imbalance intensifies, perceived work pressure also increases accordingly (Siegrist and Li, 2016). Considering that study pressure and work pressure have obvious similarities in terms of their characteristics, it can be reasonably inferred that effort–reward imbalance not only affects an individual’s perceived stress in the work scenario but can also be applied to an individual’s learning environment (Li et al., 2010; Zhang et al., 2023). From these findings, it can be inferred that the effort–reward imbalance can predict teenagers’ perceived stress. Related empirical research has also revealed that when the degree of effort–reward imbalance experienced by students during the learning process is greater, the degree of learning pressure is greater (Fukuda et al., 2010; Guo et al., 2014; Li et al., 2010). These results supported the predictive effect of effort–reward imbalance on teenagers’ perceived stress.

In addition, the stress cognitive evaluation theory indicates that when individuals encounter learning situations, they first conduct a primary evaluation to determine whether the situation poses a threat or a challenge or is irrelevant. If it is rated as a threat, a sense of stress will arise. A secondary evaluation is subsequently conducted to assess whether the available resources (time, capabilities, social support, etc.) are sufficient to address the threat. When the perceived demand exceeds the available resources, that is, when an individual’s available resources are insufficient to cope with the stressful situation they are facing, the individual enters the stress response stage, generating core dimensions of academic burnout such as emotional depletion and cynical attitudes (Lazarus and Folkman, 1984). Therefore, it can be inferred that perceived stress can predict academic burnout among teenagers. The predictive effect of perceived stress on academic burnout has also been supported by many empirical studies (Capkova, 2023; Gao, 2023; Jiang et al., 2021; Qin et al., 2020; Zhang et al., 2025). For instance, Capkova (2023) conducted a regression analysis in a sample of transnational students and reported that the explanatory rate of perceived stress for academic burnout reached 34%. Even after control variables such as emotion regulation strategies were added, the direct predictive effect of perceived stress remained significant. Therefore, based on the above analysis, Hypothesis H3 can be deduced:

*Hypothesis H*3: Perceived stress plays a mediating role between effort–reward imbalance and academic burnout.

Moreover, relevant research has shown that frustration can significantly and positively predict perceived stress (Barański and Poprawa, 2024). When an individual feels frustrated, he or she will have certain negative evaluations of the environment and tasks he or she is currently facing, believing that the learning tasks are uncontrollable or difficult to solve, which will significantly increase his or her perceived stress (Zeng et al., 2024). On the basis of the analysis of Hypotheses H2 and H3, this study can further deduce.

*Hypothesis H*4: Frustration and perceived stress play a chain mediating effect between effort–reward imbalance and academic burnout.

**Figure 1:**
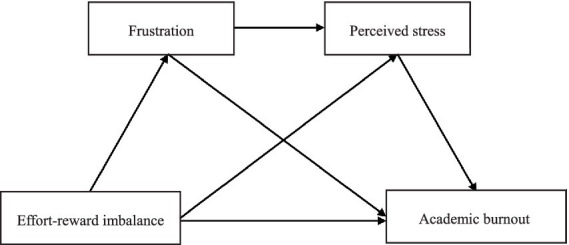
Hypothesis model diagram.

Given that how effort–reward imbalance influence adolescents’ academic burnout remains unclear, and none study investigated the relationship between effort–reward imbalance and academic burnout from the dimensions of both frustration and perceived stress, the present study aimed simultaneously test frustration and perceived stress in a chain mediation model in a large adolescent sample to reveal the influence mechanism of the effort–reward imbalance on academic burnout. On the basis of the effort–reward imbalance model and related research, this study proposes the following model (see Figure 1) to reveal the influence mechanism of effort–reward imbalance on academic burnout among teenagers from the perspective of both frustration and perceived stress.

## 2 Research methods

### 2.1 Participants

A total of 1,402 middle school students (681 boys and 721 girls) were collected with a convenience sampling method from 7 junior and senior high schools in Zhejiang Province, China. Among which 262 students were in Grade 7 (126 boys and 136 girls), 272 were in Grade 8 (134 boys and 138 girls), 66 were in Grade 9 (34 boys and 32 girls), 691 were in Grade 10 (342 boys and 349 girls), 70 were in Grade 11 (34 boys and 36 girls), and 41 were in Grade 12 (11 boys and 30 girls).

### 2.2 Measurement

#### 2.2.1 Effort–reward imbalance scale

The Effort–Reward Imbalance Scale (ERIS), developed by Fukuda et al. (2010) and revised by Chu et al. (2015), was employed. The “Effort” and “Reward” subscales were selected to assess participants’ perceived effort–reward imbalance. Using the binary scoring method, participants are required to answer a given statement by choosing either “1 point (no)” or “2 points (yes).” The “Effort” subscale consists of three questions, such as “I will strive to perform well in class.” The “Reward” subscale consists of four questions, such as “I often receive encouragement from friends at school.” The higher the score of the effort subscale is, the more effort and time the individual has invested in the learning process. The lower the score of the return subscale is, the less the individual receives a return. Effort–reward imbalance = Effort Score / (Reward Score × C), where C is the adjustment coefficient (number of questions in the effort dimension divided by the number of questions in the reward dimension). In this study, C = 0.75. Higher scores indicate a greater degree of effort–reward imbalance. The Effort–reward imbalance Scale demonstrates good reliability and validity and has been widely applied in relevant research fields (Liu et al., 2024; Wang et al., 2023).

#### 2.2.2 The defeat scale

The Defeat Scale, revised by Tang et al. (2019) and based on Gilbert and Allan (1998), was used to assess individuals’ subjective feelings of frustration and failure over the past 7 days. This scale consists of 16 items (for example, “I feel I have achieved nothing”) and uses the Likert 5-point scoring method, where “1″ indicates “completely disagree” and “5″ indicates “completely agree.” The higher the score is, the stronger the individual’s sense of frustration. This scale demonstrates good reliability and validity and has been widely applied in relevant research fields (Hu et al., 2023; Zeng et al., 2025; Zeng et al., 2024). In this research, the Cronbach’s *α* coefficient for this scale was 0.94.

#### 2.2.3 Perceived stress scale

The Chinese version of the PSS-4 was used to assess individuals’ perceived stress. This scale is a simplified version of the Perceived Stress Scale (PSS) developed by Cohen et al. (1983). It consists of four items and uses a 5-point Likert scoring method, where 1 indicates “strongly disagree” and 5 indicates “strongly agree.” Two items in the scale are reverse scored. After reverse processing, a higher total score across all the items indicated greater perceived stress among the participants. This scale demonstrates good reliability and validity and has been widely applied in relevant research fields (Carney et al., 2000; Fu et al., 2023). In this study, the Cronbach’s *α* coefficient for the PSS-4 scale was 0.72.

#### 2.2.4 Academic burnout scale

The Academic Burnout Scale for Teenagers, developed by Wu et al. (2010), was used to measure students’ academic burnout. This scale comprises three subscales—Physical and Mental Fatigue, Academic Disengagement, and Low Sense of Accomplishment—with a total of 16 items. The Physical and Mental Fatigue subscale includes 4 items, such as “I have recently felt mentally empty and do not know what to do.” The Academic Disengagement subscale comprises 5 items, such as “My academic performance is poor, and I truly want to give up”; the Low Sense of Accomplishment subscale includes 7 items, such as “I can study with high energy.” A 5-point Likert scale is used, where 1 indicates “Strongly disagree” and 5 indicates “Strongly agree.” The total score for academic burnout is calculated by summing the scores of all the items, with a higher total score indicating a greater degree of academic burnout. The Academic Burnout Scale has good reliability and validity and has been widely applied in relevant research fields (Wang et al., 2023; Yin et al., 2022). In this research, the Cronbach’s α coefficient for this scale was 0.85.

### 2.3 Procedure

This study was conducted in accordance with the Declaration of Helsinki and with local institutional/legal requirements. The research protocol was approved by the Medical Ethics Committee of Huzhou University. The selection of participants adopted a convenience sampling method, and the participants were students from 7 junior and senior high schools in Zhejiang Province, China. Before filling out the questionnaire, the informed consent form participants and their main guardians. Once they agreed to voluntarily participate in our survey, the survey questionnaire was presented to students through Questionnaire Star, and they were asked to carefully fill out the questionnaires. This survey was conducted from March 6th to March 20th, 2024. Questionnaires were distributed to students through the Questionnaire Star platform.

### 2.4 Data processing and analysis

Descriptive statistical analysis and correlation analysis were conducted using SPSS 26.0 software, while the chained mediation model was tested with the PROCESS 4.1 program. Pearson correlations, multiple linear regressions with control variables, and 95% bootstrap CIs with 5,000 samples were used to assess the confidence intervals of the mediation effects and the significance of the indirect effects.

## 3 Results analysis

### 3.1 Common method bias test

This study employed the Harman one-factor test for common method variance using SPSS 26.0. The results show that there are a total of 7 factors with eigenvalues greater than 1, and the variance interpretation rate of the first factor is 33.36% (lower than the critical index of 40%), which indicates that there is no serious common method bias problem in this study (Zhou and Long, 2004).

### 3.2 Descriptive statistics and correlation analysis of each variable

Descriptive statistics and correlation analyses were conducted using SPSS 26.0 for gender, grade, effort–reward imbalance, frustration, perceived stress, and academic burnout. For statistical convenience, boys are encoded as 1, girls as 2, and middle school grades 7 through 12 are coded as 1 through 6. The results revealed significant correlations between effort–reward imbalance, frustration, perceived stress, and academic burnout. Furthermore, gender was significantly positively correlated with frustration, perceived stress, and academic burnout, whereas grade was significantly positively correlated with effort–reward imbalance, perceived stress, and academic burnout (see Table 1).

**Table 1:** Descriptive statistics and correlation analysis of each variable.

Variable	*M*	*SD*	1	2	3	4	5	6
1. Gender	1.51	0.50	—					
2. Grade	3.11	1.37	0.02	—				
3. ERI	0.94	0.26	−0.02	0.09**	—			
4. Frustration	2.52	0.84	0.08**	0.04	0.39**	—		
5. Perceived stress	2.76	0.77	0.10**	0.14**	0.35**	0.67**	—	
6. Academic burnout	2.80	0.63	0.12**	0.24**	0.38**	0.67**	0.71**	—

ERI, effort–reward imbalance; **p < 0.01.

### 3.3 Chain mediation model testing

In this study, Model 6 from the PROCESS 4.1 plugin for SPSS 26.0 developed by Hayes (2017) was employed to examine the mediating effect of frustration and perceived stress on the relationship between effort–reward imbalance and academic burnout. The bootstrap method was used for 5,000 repeated samplings to determine the significance of the mediating effect and calculate the 95% confidence interval. Considering the significant positive correlations between gender and frustration, perceived stress, and academic burnout, as well as between grade and effort–reward imbalance, perceived stress, and academic burnout, we controlled for gender and grade when testing the chain mediation model.

The results revealed that before the mediating variables were included, the effort–reward imbalance significantly and positively predicted academic burnout (*β* = 0.367, *p* < 0.001), and the 95% confidence interval was [0.7644, 0.9892]. After controlling for the mediating variables, the direct predictive effect of effort–reward imbalance on academic burnout remained significant (*β* = 0.090, *p* < 0.001). The predictive effects of effort–reward imbalance on frustration (*β* = 0.396, *p* < 0.001) and perceived stress (*β* = 0.090, *p* < 0.001) were also significant. Moreover, frustration significantly and positively predicted perceived stress (*β* = 0.629, *p* < 0.001) and academic burnout (*β* = 0.333, *p* < 0.001). Additionally, perceived stress significantly and positively predicted academic burnout (*β* = 0.425, *p* < 0.001; see Table 2). The results of the preliminary analysis of the mediating effect reveal that the mediating effect of frustration and perceived stress between effort–reward imbalance and academic burnout is established. Moreover, regardless of whether the mediating variables were included, effort–reward imbalance could significantly predict academic burnout, indicating that frustration and perceived stress have a partial mediating effect on the relationship between effort–reward imbalance and academic burnout.

**Table 2:** Chain mediation model testing.

Result variables	Predictors	*R* ^2^	*F*	*β*	*t*
Frustration	Effort–reward imbalance	0.163	90.788***	0.396	16.120***
	Gender			0.087	3.571***
	Grade			0.001	0.024
Perceived stress	Effort–reward imbalance	0.473	313.650***	0.090	4.248***
	Frustration			0.629	29.606***
	Gender			0.051	2.622**
	Grade			0.104	5.318***
Academic burnout	Effort–reward imbalance	0.602	422.463***	0.090	4.858***
	Frustration			0.333	14.160***
	Perceived stress			0.425	18.268***
	Gender			0.050	2.962**
	Grade			0.161	9.415***

**p < 0.01; ***p < 0.001.

**Figure 2:**
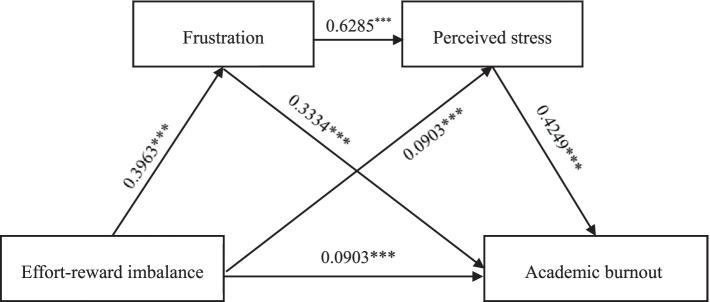
The chain mediating effect of frustration and perceived stress on the relationship between effort–reward imbalance and academic burnout.

In this study, the mediating path mechanism through which effort–reward imbalance predicts academic burnout consists of the following three paths: The first is a single mediating path of effort–reward imbalance→ frustration→ academic burnout; the second is a single mediating path of effort–reward imbalance→ perceived stress→ academic burnout; and the third is a chain mediating pathway: effort–reward imbalance→ frustration→ perceived stress→ academic burnout. To further analyze the essence of the mediating effect of frustration and perceived stress on the relationship between effort–reward imbalance and academic burnout, we conducted an in-depth analysis of the mediating pathway. The results revealed that the indirect effect value of the mediating path of effort–reward imbalance→ frustration→ academic burnout was 0.132, with a 95% confidence interval of [0.1056, 0.1611], and the indirect effect value of the mediating path of effort–reward imbalance→ perceived stress→ academic burnout was 0.038, with a 95% confidence interval of [0.0205, 0.0573]. The indirect effect value of the chain mediating path of effort–reward imbalance→ frustration→ perceived stress→ academic burnout was 0.106, with a 95% confidence interval of [0.0864, 0.1263]. In this study, the 95% confidence intervals of the three indirect effects did not include 0, indicating that both the single mediating effect and the chain mediating effect of frustration and perceived stress were significant. The results further confirm that frustration and perceived stress mediate the relationship between effort–reward imbalance and academic burnout (see Figure 2; Table 3).

**Table 3:** Path analysis of the chain mediating effect.

Effect type	Effect	se	*t*	LLCI	ULCI	Relative effect value
Total effect	0.3666	0.0171	15.30***	0.7644	0.9892	
Direct effect	0.0903	0.0445	4.86***	0.1288	0.3032	24.63%
Ind 1	0.1321	0.0142	/	0.1051	0.1605	47.81%
Ind 2	0.0384	0.0096	/	0.0197	0.0574	13.90%
Ind 3	0.1058	0.0142	/	−0.0959	−0.0403	38.29%

Ind 1 = ERI → frustration → burnout; Ind 2 = ERI → stress → burnout; Ind 3 = ERI → frustration → stress → burnout; the reported coefficients are standardized. ***p < 0.001.

Further comparative analysis of the mediating effect shows that the mediating effect through frustration (Ind 1 = 0.1312) is significantly greater than that through perceived stress (Ind 2 = 0.0384), with a difference value of 0.2242, and the 95% confidence interval is [0.1345, 0.3171]. The mediating effect through both frustration (Ind 1 = 0.1312) and that through perceived stress (Ind 2 = 0.0384) were not significantly different from the chain mediation effect (Ind 3 = 0.1058).

## 4 Discussion

Academic burnout has a significant negative effect on teenage students’ academic performance, learning motivation, and even their physical and mental health (Madigan and Curran, 2021; Schaufeli et al., 2002; Wang et al., 2015). Existing research has investigated the mechanisms underlying academic burnout from the dimensions of individual characteristics (Yu et al., 2023), family background factors (Yin et al., 2022), and school educational environments (Luo et al., 2014). However, research on the relationship between effort–reward imbalance and teenagers’ academic burnout, as well as its underlying mechanisms, remains scarce. Whether and how effort–reward imbalance influences academic burnout among teenagers needs further clarification. On this basis, this study examined the predictive effect and mechanism of the effort–reward imbalance on teenagers’ academic burnout from the two perspectives of frustration and perceived stress. The results indicated that frustration and perceived stress partially mediated the relationship between effort–reward imbalance and academic burnout. Next, we discuss the results of this research in terms of the predictive effect of the effort–reward imbalance on academic burnout, the mediating effect of frustration, and the mediating effect of perceived stress.

### 4.1 The predictive effect of effort–reward imbalance on academic burnout

This research first examined the ability of effort–reward imbalance to predict academic burnout among teenagers. The results indicated that effort–reward imbalance significantly and positively predicted academic burnout among teenagers, regardless of whether mediating variables were included. Specifically, the greater the degree of effort–reward imbalance, the higher the level of academic burnout experienced by teenagers in learning activities. Hypothesis H1 hypothesized that the effort–reward imbalance could significantly predict teenagers’ academic burnout, according to the effort–reward imbalance model. These findings align with the model predictions, further validating and reinforcing the applicability of the effort–reward imbalance model. This discovery also concurs with existing empirical research (Gao et al., 2025; Jin et al., 2025; Wang et al., 2023), indicating that effort–reward imbalance is a crucial factor influencing teenagers’ academic burnout. This study, while verifying the model’s predictions, has expanded our understanding of the factors affecting academic burnout among teenagers and provides important guidance for educational practice. These findings encourage educators to give close attention to the degree of effort–reward imbalance among teenagers during their learning process. By taking reasonable teaching and support measures, educators can help avoid or reduce teenagers’ sense of effort–reward imbalance, thereby effectively mitigating the negative impact of academic burnout.

### 4.2 Mediating effect of frustration

After confirming the predictive effect of effort–reward imbalance on teenagers’ academic burnout, we further examined the mediating effect of frustration between effort–reward imbalance and academic burnout. On the basis of frustration theory (Miller, 1941) and self-determination theory (Deci and Ryan, 2000; Ryan and Deci, 2000), as well as empirical research related to effort–reward imbalance and frustration and to frustration and academic burnout (Guo et al., 2014; Li et al., 2010; Fariborz et al., 2019; Wang et al., 2023; Zhang and Jiang, 2023), we deduced the Hypothesis H2, in which we hypothesize that frustration mediates the relationship between effort–reward imbalance and academic burnout. By testing for mediating effects in the survey data, we found that frustration partially mediated the relationship between effort–reward imbalance and academic burnout. These findings not only validate our hypothesis but also further support the results of frustration theory, self-determination theory, and related empirical research. Moreover, the findings of this study elucidate the psychological mechanism through which effort–reward imbalance influences teenagers’ academic burnout from a frustration perspective. This study deepens our understanding of the mechanism through which effort–reward imbalance affects academic burnout among teenagers and offers certain insights for educational practice. In educational practice, educators should simultaneously give attention to the degree of effort–reward imbalance in teenagers’ learning activities and the resulting frustration. When the effort–reward imbalance experienced by teenagers is difficult to completely avoid, preventive interventions, such as providing emotional support and reinforcing positive feedback, can mitigate the negative impact of the effort–reward imbalance on teenagers’ frustration, thereby indirectly reducing the risk of academic burnout.

### 4.3 Mediating effect of perceived stress

After further confirming the partial mediating effect of frustration, we proceeded to examine the mediating effect of perceived stress between effort–reward imbalance and academic burnout and further investigated the chain mediating effect of frustration and perceived stress between effort–reward imbalance and academic burnout. Relying on the new ternary effort–reward imbalance model (Siegrist and Li, 2016), the cognitive appraisal theory of stress, and empirical research on the correlations between effort–reward imbalance and perceived stress, as well as between perceived stress and academic burnout (Capkova, 2023; Fukuda et al., 2010; Gao, 2023; Guo et al., 2014; Jiang et al., 2021; Lazarus and Folkman, 1984; Li et al., 2010; Qin et al., 2020; Zhang et al., 2025), we deduced the Hypothesis H3, in which we hypothesize that perceived stress mediates the relationship between effort–reward imbalance and academic burnout. Through a mediation analysis of survey data, we found that perceived stress partially mediated the relationship between effort–reward imbalance and academic burnout. Furthermore, the research results also revealed that the mediating effect through frustration was significantly greater than that through perceived stress. These findings not only validate Hypothesis H3 but also further support the effort–reward imbalance model, the cognitive appraisal theory of stress, and conclusions from related empirical research. More importantly, the findings of this research further elucidate the psychological mechanisms through which effort–reward imbalance influences teenagers’ academic burnout from a perceived stress perspective and the differences in the mediating effect between perceived stress and frustration. This deepens our own understanding of the mechanisms of action through which effort–reward imbalance affects academic burnout among teenagers and provides valuable inspiration for educational practice activities. Educators should recognize that, in educational practice, while giving attention to the effort–reward imbalance among teenagers and the frustration it causes, they must also consider how this effort–reward imbalance affects teenagers’ perceived stress. When such effort–reward imbalance is inevitable, some preventive interventions can be implemented, such as systematically teaching stress management skills, to mitigate the negative impact of effort–reward imbalance on teenagers’ perceived stress to indirectly curb their academic burnout.

Moreover, through chained mediation analysis of survey data, we found that frustration and perceived stress not only mediate the relationship between effort–reward imbalance and academic burnout through a single pathway but also mediate this relationship through a chained pathway of frustration and perceived stress. These findings also offer meaningful inspiration for our educational practices. These findings suggest that educators should adopt comprehensive measures to mitigate the direct negative impact of effort–reward imbalance on teenagers’ frustration and perceived stress, as well as its indirect negative influence on academic burnout. This approach aims to minimize the risk of academic burnout and provide a more robust psychological foundation for teenagers’ healthy physical and mental development.

## 5 Contributions, limitations and future research

This research tested the predictive effect of effort–reward imbalance on teenagers’ academic burnout from the perspectives of frustration and perceived stress. The results revealed that frustration mediated the relationship between effort–reward imbalance and academic burnout, whereas perceived stress also mediated the relationship between effort–reward imbalance and academic burnout. In addition, the chain mediating effect of frustration and perceived stress on the relationship between effort–reward imbalance and academic burnout was also tested. The results not only further supported several previous classic theories, such as the effort–reward imbalance model, frustration theory, self-determination theory and the cognitive appraisal theory, but also revealed the psychological mechanism through which effort–reward imbalance influences teenagers’ academic burnout and enhanced people’s understanding on the prediction mechanism of the effort–reward imbalance on academic burnout.

However, the following limitations still require further exploration in follow-up research. First, this research adopted cross-sectional data to test our hypothesized model. While it demonstrated the correlations among the variables, it was difficult to provide strong evidence for the causality between them. Second, the mechanism of action through which effort–reward imbalance affects teenagers’ academic burnout is complicated and diverse. Moreover, there are many protective factors that can buffer the negative impact of effort–reward imbalance on academic burnout, like social support, peer support, teacher support, and parent support. This research explored this topic only from the two perspectives of frustration and perceived stress, but did not explore how those protective factors buffer the negative impact of effort–reward imbalance on academic burnout.

Therefore, future research could (1) try to collect longitudinal tracking data or design experimental interventions to fully validate and reveal the causal relationships among effort–reward imbalance, frustration, perceived stress, and academic burnout; (2) consider further investigating the influence mechanism of effort–reward imbalance on teenagers’ academic burnout from more perspectives.

## 6 Conclusion

This research, which is grounded in frustration theory, self-determination theory, the new ternary effort–reward imbalance model, and stress cognitive evaluation theory, along with related empirical research, proposes a structural equation model for predicting teenagers’ academic burnout through effort–reward imbalance, which is viewed from the perspectives of frustration and perceived stress. The model was tested using cross-sectional data. The research yields the following conclusions: (1) Effort–reward imbalance positively predicts academic burnout among teenagers, and (2) frustration and perceived stress mediate the relationship between effort–reward imbalance and academic burnout. Specifically, both frustration and perceived stress can mediate this relationship through a single mediating pathway or a chain mediating pathway of frustration and perceived stress. The research simultaneously tests frustration and perceived stress as chain mediators of ERI in adolescent burnout, the results not only validate relevant theories and enhance people’s understanding on the prediction mechanism of the effort–reward imbalance on academic burnout, but also provide valuable insights for educational practice. Of course, this study also has some flaws that need to be further improved in future research.

## Data Availability

The datasets presented in this study can be found in online repositories. The names of the repository/repositories and accession number(s) can be found in the article/supplementary material.
